# Observational Study of Glycemic Impact of Anticipatory and Early-Race Athletic Competition Stress in Type 1 Diabetes

**DOI:** 10.3389/fcdhc.2022.816316

**Published:** 2022-05-03

**Authors:** Nicole Hobbs, Rachel Brandt, Sadaf Maghsoudipour, Mert Sevil, Mudassir Rashid, Laurie Quinn, Ali Cinar

**Affiliations:** ^1^ Department of Biomedical Engineering, Illinois Institute of Technology, Chicago, IL, United States; ^2^ Department of Chemical and Biological Engineering, Illinois Institute of Technology, Chicago, IL, United States; ^3^ College of Nursing, University of Illinois at Chicago, Chicago, IL, United States

**Keywords:** anxiety, athlete, physical activity, stress, type 1 diabetes

## Abstract

Athletic competitions and the associated psychological stress are a challenge for people with type 1 diabetes (T1D). This study aims to understand the influence of anticipatory and early race competition stress on blood glucose concentrations and to identify personality, demographic, or behavioral traits indicative in the scope of the impact. Ten recreational athletes with T1D competed in an athletic competition and an exercise-intensity matched non-competition “training” session for comparison. The two hours prior to exercise and the first 30 minutes of exercise were compared between the paired exercise sessions to assess the influence of anticipatory and early race stress. The effectiveness index, average CGM glucose, and the ingested carbohydrate to injected insulin ratio were compared between the paired sessions through regression. In 9 of 12 races studied, an elevated CGM for the race over the individual training session was observed. The rate of change of the CGM during the first 30 minutes of exercise notably differed between the race and training (p = 0.02) with a less rapid decline in CGM occurring during the race for 11 of 12 paired sessions and an increasing CGM trend during the race for 7 of the 12 sessions with the rate of change (mean ± standard deviation) as 1.36 ± 6.07 and -2.59 ± 2.68 mg/dL per 5 minutes for the race and training, respectively. Individuals with longer durations of diabetes often decreased their carbohydrate-to-insulin ratio on race day, taking more insulin, than on the training day while the reverse was noted for those newly diagnosed (r = -0.52, p = 0.05). The presence of athletic competition stress can impact glycemia. With an increasing duration of diabetes, the athletes may be expecting elevated competition glucose concentrations and take preventive measures.

## 1 Introduction

In the management of type 1 diabetes (T1D), exercise can represent a significant challenge as the glycemic response to exercise is influenced by the exercise intensity and duration, the composition and timing of prior meals or snacks, the amount of circulating insulin, and the location of insulin delivery (1). Individuals with T1D need to adjust their insulin administration and carbohydrate consumption to accommodate these factors. Experts in exercise physiology and endocrinology have developed guidelines to help people with T1D to exercise and compete in athletic competitions safely (1–3), and these broad recommendations are a foundation for the treatment plan that needs to be adjusted based upon the current physiological, psychological and metabolic state of the individual. This challenge is amplified on competition days, as the added stress can cause drastically different glucose responses than on training days.

The existing research on athletic competitions with people with T1D has focused on demonstrating the relative safety of participation and to acknowledge that a subpopulation of competitive athletes with T1D exists ranging from recreational (4, 5) to professional athletes (3). For example, T1D Olympic swimmer Gary Hall, Jr. reported that his blood glucose can spike from 100 mg/dL to 300 mg/dL in the 21 seconds of a 50 meter race (6). This spike in blood glucose concentrations (BGC) likely occurred due to a combination of increased hepatic glycogenolysis associated with high-intensity physical activity and the hormone responses, such as epinephrine, glucagon, growth hormone and cortisol, associated with the stress of competition (7). Competition anxiety has been highly studied in terms of performance and the personal psycho-social factors that influence its presence (8), but its influence on BGC in people with T1D has not been previously studied.

The anxiety response is highly dependent on an individual’s personality traits. Obtaining feedback on each individual’s general perceptions of anxiety can provide insight into the role that anxiety may play in competitive sports. Anxiety can be assessed through surveys or measurement of relevant physiological variables (9, 10). While it is widely accepted that athletes with T1D should expect competition anxiety to influence their blood glucose dynamics, the degree to which these changes occur and the relationship of these changes to the amount of anxiety has not been well studied. A better understanding of this response will increase safety for athletes with T1D and may improve their athletic performances.

The goal of this research study was two-fold. In a group of adults with T1D, we observed the influence of anticipatory and early race athletic competition stress on BGC and identified the personality, demographic, or behavioral traits that impacted this stress. This was the first study to consider the influence of athletic competition stress through a comparison of the athletic competition and a non-competitive intensity-matched exercise session. The results of this study may impact the advice given to recreational athletes with T1D to optimize their BGC to increase safety and help them to achieve their best athletic performances.

## 2 Materials and Methods

### 2.1 Experimental Setup and Inclusion Criteria

Ten individuals (aged 18-60) with a diagnosis of T1D for greater than 6 months and planned athletic competition within the study period were recruited for participation in this study. These individuals were required to have completed a similar athletic competition within the last two years and must have been following the same diabetes therapy at that time without a severe hypo- or hyperglycemic event requiring assistance from a medical professional. Subjects were excluded from the study for the following reasons: metabolic instability as evidenced by hospitalizations for diabetes or other diabetes-related complications (e.g., diabetic ketoacidosis and hypoglycemic seizures) within the preceding three months; severe macrovascular disease, as evidenced by severe peripheral artery disease (PAD; e.g., tissue ischemia with/at risk for gangrene and amputation); history of myocardial infarction, heart failure, thromboembolic disease, or unstable angina; uncontrolled hypertension; severe microvascular disease as evidenced by history of vision-threatening proliferative or non-proliferative retinal disease; kidney disease; any uncontrolled non-musculoskeletal condition that would limit the subject’s ability to participate in the exercise program (e.g., chronic obstructive airways disease); musculoskeletal conditions such as neurological or orthopedic conditions affecting lower limb strength and mobility (e.g., stroke; insensitive foot); pregnancy; and documented medical condition or physical impairment that is judged by the health care practitioner to contraindicate exercise. This study was approved by the Illinois Institute of Technology Institutional Review Board.

### 2.2 Details of Procedures

This was an observational descriptive study with the subjects following their standard physical activity routines at home and participating in an athletic competition of their choosing (running race ranging from 5K to marathon distance). For 12 hours prior to the competition, the subjects kept detailed diaries about their meals, snacks, insulin doses, and physical activity (type, time, duration, intensity). A continuous glucose monitor (CGM) (Dexcom G6, San Diego, CA) recorded the glycemic responses. To measure physiological variables of interest, a wristband (Empatica E4, Milan, Italy) (11) was worn for the competition if the subject was willing. The wristband has a photoplethysmography (PPG) sensor that generates heart rate and heart rate variability, an infrared thermopile to read peripheral skin temperature, an electrodermal activity sensor, and a 3-dimensional accelerometer.

The subjects completed a second exercise session which closely mimicked their competition exercise session in terms of intensity. In this non-competition session, the subjects completed 30 minutes of running at their competition pace. For 12 hours prior to this non-competitive exercise session, the subjects were asked to consume the same meals or snacks, insulin dosing if applicable, feasible and would not impair health, and any routines they have for preparation for the competition. The subjects kept detailed diaries about their meals, snacks, insulin doses, and physical activity (type, time, duration, intensity). The wristband and CGM were provided for physiological signal and glucose measurements.

A brief health history was performed, hemoglobin A1C (A1CNow+; Bayer, Metrika, Sunnyvale, CA) was obtained, and subjects completed the State-Trait Anxiety Inventory (STAI) (9) and the Sport Competition Anxiety Test (SCAT) (8) to assess anxiety-proneness in general and in relation to athletic competitions. Trait anxiety is a personality trait representative of relatively stable individual differences in anxiety-proneness. Thus, a person’s tendency to perceive a situation as stressful, dangerous, or threatening is related to their trait anxiety. The participants also completed the Hypoglycemia Fear Survey II (HFS) (12) to assess the influence of fear of hypoglycemia on diabetes management behaviors surrounding the competitive and non-competitive exercise sessions.

Ten people with type 1 diabetes completed the study. Two individual participated in the study twice for a total of 12 races and 12 intensity-matched non-competition exercise sessions studied.

### 2.3 Data Analysis

The difference between the competition and non-competition exercise session in CGM glucose concentrations and the slope of the CGM was assessed with student’s t-tests. The slope was determined through simple linear regression. The percentage of time above, below, and inside the target glucose range is assessed.

Regression models were developed to assess the influence of the athletic competition stress on (A) the ratio of ingested carbohydrates to injected insulin (ICII) calculated as the specific amount of insulin administered, above the basal infusion rate, relative to the reported amount of carbohydrates consumed, (B) an “effectiveness index” which quantifies the variability in glucose concentration after accommodating for the expected effect of administered insulin and carbohydrates consumed (13, 14), (C) the average CGM glucose (CGM) in the anticipatory period, (D) the average CGM in the first 30 minutes of exercise, (E) the slope of the CGM in the anticipatory period, and (F) the slope of the CGM in the first 30 minutes of exercise. Additional details regarding the calculation of these metrics can be found in the **supplementary material section 2**. These regression models predicted the difference in these metrics between the competition and non-competition exercise within each participant. The within-individual variation is of interest since a higher interpersonal variability is expected. The data included for modeling included the anticipatory stress period and the early exercise period defined as three hours prior to the exercise session through 30 minutes of exercise. The proposed models included inputs corresponding to the STAI trait-anxiety score, SCAT score, HFS, Age, Duration of Diabetes, HbA1c, and BMI.

Projection to latent structures regression (PLS), also called partial least squares, was applied to assess the interactions between the normalized input variables using the SIMPLS algorithm (15). In PLS, a latent variable is a linear combination of input variables where the weight vectors to calculate the latent variables are called loading vectors. The latent variables are oriented such that they best explain the variance in the input variables and the variance in the response variable while explaining the maximal possible variance between the input and response variables.

The significant inputs of the PLS models were used in multiple linear regression (MLR) models and assessed through the coefficient of determination, *R* ^2^, the adjusted *R* ^2^, and the predictive *R* ^2^. The predictive *R* ^2^ is a method which applies leave one out cross validation to provide a metric assessing the model fit to data that were removed from the set of data used to estimate the model. This metric is calculated as the sum of squares of the residuals of the withheld data points. A predictive *R* ^2^ value greater than 0.5 indicates a model with strong predictive ability (16).

As a secondary assessment of variables that were not significant in the regression models, Pearson’s correlation coefficient was employed to assess correlation between variables of interest with reported p-values corresponding to the student’s t-distribution.

## 3 Results

Ten subjects completed the study and two of them completed 2 competitive races each for a total of 12 races and 12 intensity-matched non-competitive exercise sessions studied. The subjects were recreational athletes with race paces ranging from 6:30 minutes per mile (4 minutes per kilometer for subject participating in a 5K race) to 11 minutes per mile (6 minutes 50 seconds per kilometer for subject participating in a marathon). These subjects represented a wide range of general trait anxiety as measured by the Trait subscale of State Trait Anxiety Inventory as demonstrated by a score of 70% [29, 89] (median [interquartile range]) percentile rank where the 50% would represent the average score for age and gender matched adults (9). These subjects comprised an average trait anxiety group relative to those individuals participating in athletic competitions as measured by the Sport Competition Anxiety test (SCAT) with scores of 22 [19, 24] where a score below 17 indicates low trait anxiety and a score greater than 24 indicates high trait anxiety in competitive athletes (8). These subjects scored slightly higher on the Hypoglycemia Fear Survey II - Behavior (HFS-B) section (21 [18, 24]) and slightly lower than average on the Hypoglycemia Fear Survey II - Worry (HFS-W) section (19 [16, 24]) than the general T1D adult population with scores of 17.9 ± 9.3 and 22.3 ± 14.4, respectively (12). The demographic information is included in **Table 1**.

**Table 1:** Demographic Information of the Athletic Competition Study Participants. Information recorded as median [interquartile range].

Demographic Information	
Gender	6 Male/4 Female
Age (years)	32 [25, 38]
Duration of Diabetes (years)	13 [3, 24]
BMI (kg/m^2^)	23.1 [21.2, 26.3]
HbA1c (%)	6.1 [6.0, 6.5]
Diabetes Treatment	5 Pump/5 MDI
Personal CGM Use	All
Race Distance	2 x 5k, 1 x 7k, 1 x 10k, 1 x 10-mile, 7 x Marathon

**Figure 1:**
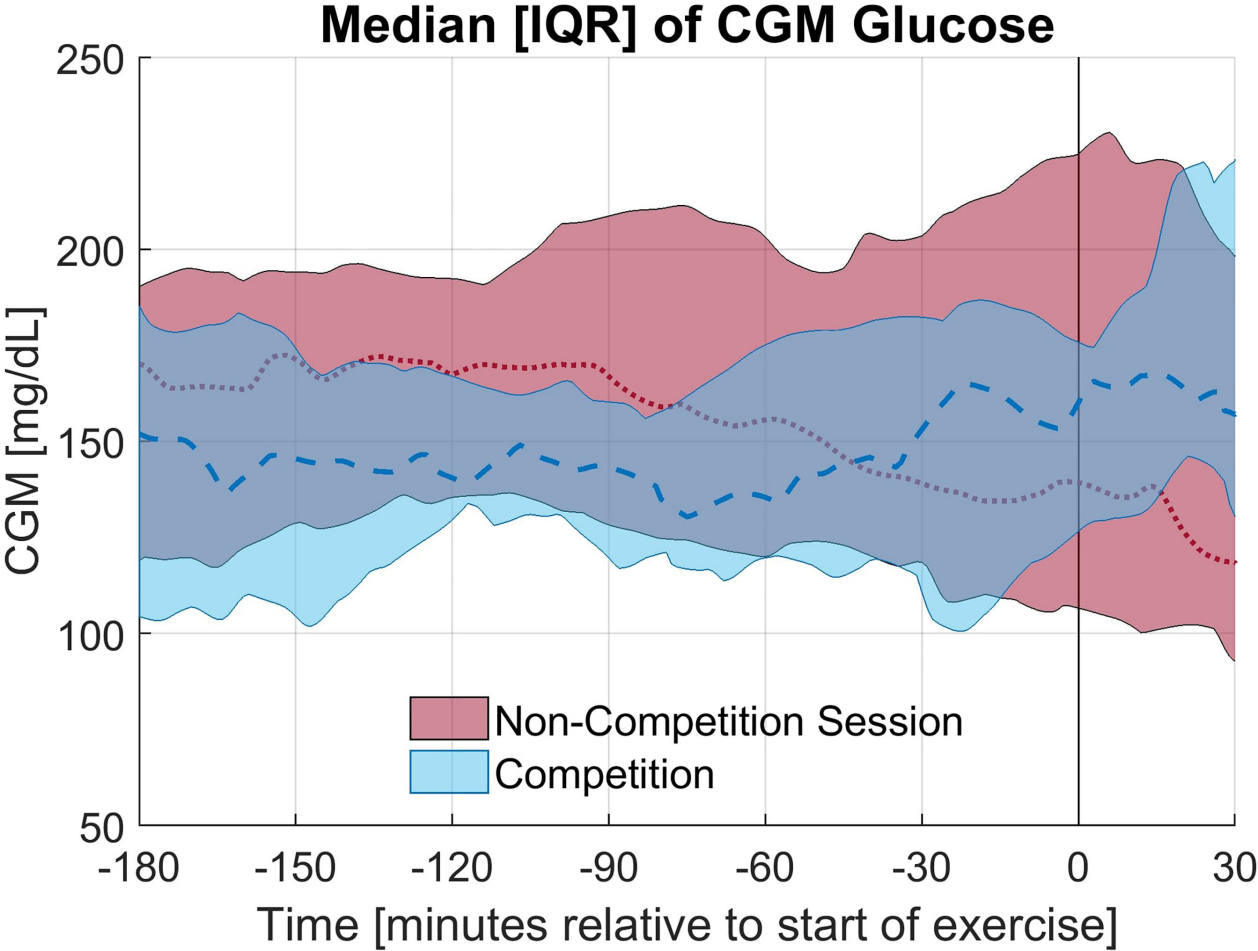
Median and Interquartile range for CGM glucose concentration for the competition and non-competition exercise sessions.

**Figure 2:**
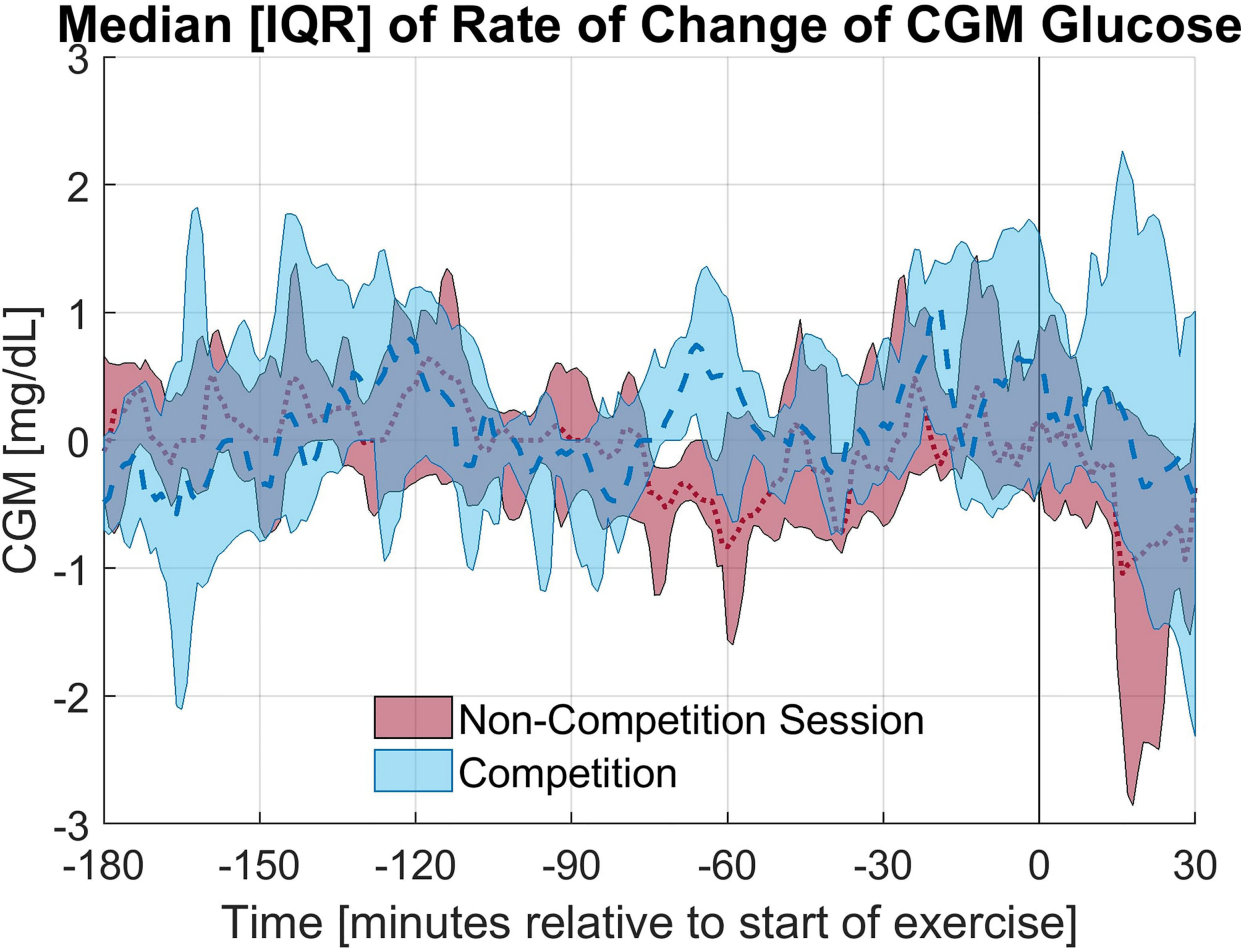
Median and Interquartile range for the rate of change of CGM glucose concentration for the competition and non-competition exercise sessions.

The exercise period was defined as the first 30 minutes of exercise. In 9 of 12 races studied, the average CGM glucose during the exercise period was elevated during the race compared to the individual training session; however, this increase was not statistically significant (p = 0.28). Alternatively, in only 4 of 12 sessions the average glucose concentration was higher in the anticipatory period (3 hours prior to exercise) on the day of the race compared to the day of the non-competition exercise session. The elevation in glucose concentration observed on the day of the race is primarily occurring in the period of study. The rate of change of the CGM during the first 30 minutes of exercise notably differed between the race and training (p = 0.02) with a less rapid decline in CGM occurring during the race for 11 of 12 paired sessions and an increasing CGM trend during the race for 7 of the 12 sessions with the rate of change (mean ± standard deviation) as 1.36 ± 6.07 and -2.59 ± 2.68 mg/dL per 5minutes for the race and training, respectively. The median and interquartile range relative to the start of exercise for CGM glucose concentration and rate of change in CGM glucose concentration for the competition and non-competition sessions are shown in **Figure 1**, **2**, respectively.

Overall, the percentage of time in the target glycemic range (70-180 mg/dL) was higher on the day of the race compared to the training session in the anticipatory period as shown in **Table 2**, but this increase was not statistically significant (p = 0.93). The difference was smaller for the exercise period, but again tighter glycemic control was observed on the day of the race. Notably, an increase in hyperglycemia (>180 mg/dL) was observed in the first 30 minutes of the race while an increase in time in euglycemia was observed on the training day. A few individuals experienced more severe hyperglycemia on the day of the race. The average glucose concentrations were similar in the anticipatory period, yet the average glucose concentrations in the first 30 minutes of exercise differed more greatly with a statistically insignificant increase observed on race day (p = 0.28).

**Table 2:** Percent of Time in Each Glycemic Range and the Average CGM Glucose by Event (median [25th quantile, 75th quantile].

CGM Range by Event		<55^1^	55 – 70	70 – 180	180 – 250	>250	Average
Race	Anticipatory (3 hours)	0 [0,0]	0 [0,0]	96.1 [64.2,100]	3.9 [0,24.5]	0 [0,0]	143.1 [127.4,162.0]
	Exercise (30 min)	0 [0,0]	0 [0,0]	93.1 [37.9,100]	0 [0,20.7]	0 [0,1.7]	167.5 [138.0,196.0]
Training	Anticipatory (3 hours)	0 [0,0]	0 [0,0]	83.1 [23.4,100]	9.1 [0,58.6]	0 [0,3.6]	156.9 [125.3,203.5]
	Exercise (30 min)	0 [0,0]	0 [0,0]	100 [12.1,100]	0 [0,24.1]	0 [0,36.2]	129.6 [100.6,223.2]

^1^mg/dL.

**Figure 3:**
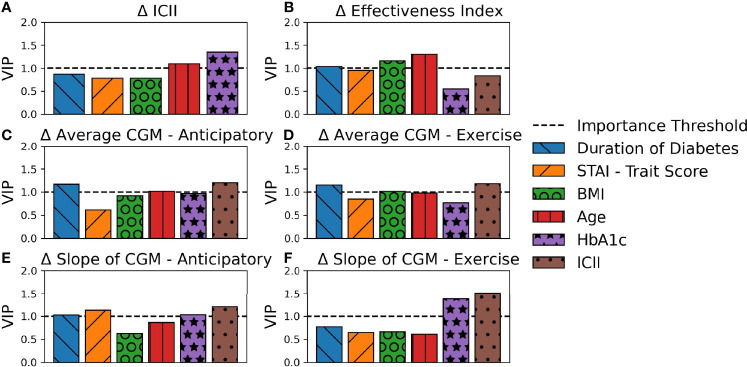
Contribution of PLS Loadings per Input Variable. The total percent variance of the response variable explained by each latent variable is indicated by total bar height. The contribution of each input variable to the corresponding latent variable is indicated by area of shading and in order of appearance with larger loadings on the top. Each subplot represents a PLS model for a unique response variable with **(A)** the difference in ICII, **(B)** the difference in Effectiveness Index, **(C)** the difference in average CGM in the anticipatory period **(D)** the difference in average CGM in the exercise period, **(E)** the difference in the slope of the CGM in the anticipatory period, and **(F)** the difference in the slope of the CGM in the exercise period.

PLS regression explained a large percentage of the variance of several of the analyzed output response variables with the models for (A) Difference in ICII Ratio (90.0%), (C) Difference in Average CGM - Anticipatory (86.5%)), (D) Difference in Average CGM - Exercise (85.0%), and (E) Difference in Slope of CGM - Anticipatory (84.5%) as shown in **Figure 3** and **Supplementary Table S3**. The PLS models captured less of the variance observed in (B) Difference in Effectiveness Index (38.2%) and (F) Difference in Slope of CGM - Exercise (50.4%) as shown in **Figure 3** and **Supplementary Table S3**. The latent variables capture the systemic variation in the data set, with each latent variable successively capturing variations that are not encoded in the preceding more dominant latent variables, thus yielding latent variables that do not capture redundant information and succinctly describe the variations in the data set. **Figure 3** illustrates the contributions of the input variables to each latent variable of the PLS models and the total variance of the response variable explained by each component.

**Figure 4:**
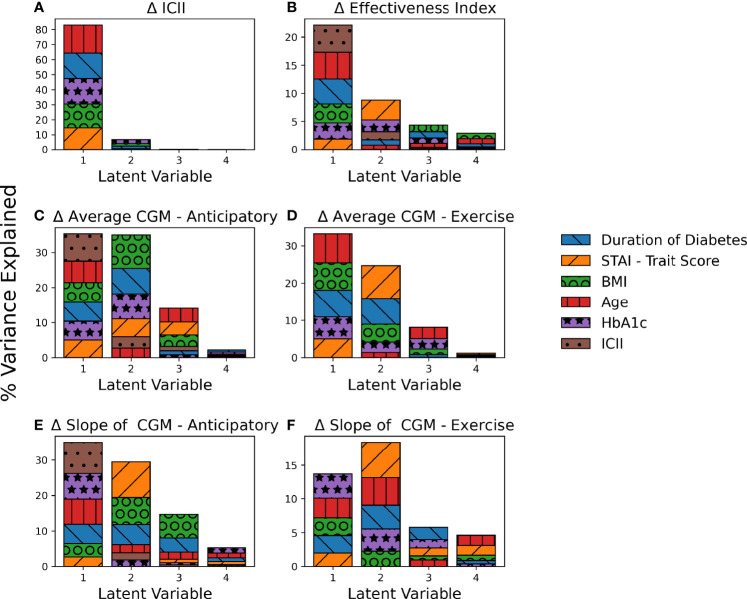
Variable importance in explaining variations observed in the response variables. Each subplot represents a PLS model for a unique response variable with **(A)** the difference in ICII, **(B)** the difference in Effectiveness Index, **(C)** the difference in average CGM in the anticipatory period **(D)** the difference in average CGM in the exercise period, **(E)** the difference in the slope of the CGM in the anticipatory period, and **(F)** the difference in the slope of the CGM in the exercise period.

The behavioral variable of the difference in ICII between the race and training session was highly explained by the included demographic data (**Figure 3A**). The variables with the most significant relationship to the difference in the ingested carbohydrate to injected insulin ratio between the training and competition sessions include HbA1c and age (**Figure 4A**). In the corresponding MLR model, **Table 3A**, both age and HbA1c are significant variables and the model explains a high degree of variance in the observed data with *R* ^2^=0.86, adjusted *R* ^2^=0.83, and p < 0.001 and the relationship is likely to be representative of new data points due to the high predictive *R* ^2^ as shown in **Table 4A**.

**Table 3:** Significant Variables in the Multiple Linear Regression Models of the Difference between Competition and Non-competition Data.

Multiple Linear Regression Models	(A) Difference in ICII Ratio	
Parameter	Coefficient [95% CI]	p-value
Intercept	-71.1 [-125.8,-16.5]	0.016
Age	-1.0 [-1.6, -0.5]	0.003
HbA1c	17.1 [9.6, 24.5]	0.001
**Response Variable**	**(B) Difference in Effectiveness Index**	
**Parameter**	**Coefficient [95% CI]**	**p-value**
Intercept	1070.1 [-1239.3,3379.5]	0.3165
Age	-24.7 [-72.8, 23.5]	0.2717
Duration of Diabetes	2.2 [-37.1, 41.6]	0.8987
BMI	-25.6 [-113.7, 62.5]	0.5219
**Response Variable**	**(C) Difference in Average CGM - Anticipatory**	
**Parameter**	**Coefficient [95% CI]**	**p-value**
Intercept	-555.6 [-896.5,-214.8]	0.006
Age	1.7 [-2.9, 6.2]	0.421
Duration of Diabetes	-4.2 [-7.4, -1.1]	0.016
HbA1c	91.5 [29.7, 153.4]	0.010
ICII	-5.3 [-8.4, -2.2]	0.005
**Response Variable**	**(D) Difference in Average CGM - Exercise**	
**Parameter**	**Coefficient [95% CI]**	**p-value**
Intercept	-334.5 [-749.7,80.7]	0.098
Age	6.6 [-1.2,14.4]	0.086
Duration of Diabetes	-4.6 [-10.0, 0.7]	0.081
BMI	9.6 [-3.7,22.8]	0.131
ICII	-0.8 [-3.7, 2.0]	0.515
**Response Variable**	**(E) Difference in Slope of CGM - Anticipatory**	
**Parameter**	**Coefficient [95% CI]**	**p-value**
Intercept	-4.28 [-8.24,-0.32]	0.038
Duration of Diabetes	-0.05 [-0.08,-0.02]	0.008
STAI - Trait	0.04 [0.02, 0.07]	0.003
HbA1c	0.57 [-0.09, 1.22]	0.080
ICII	-0.06 [-0.10,-0.03]	0.002
**Response Variable**	**(F) Difference in Slope of CGM - Exercise**	
**Parameter**	**Coefficient [95% CI]**	**p-value**
Intercept	0.11 [-11.01,11.24]	0.982
HbA1c	0.09 [-1.71, 1.89]	0.911
ICII	-0.16 [-0.61, 0.29]	0.430
HbA1c x ICII	0.03 [-0.04, 0.09]	0.352

**Table 4:** Model Fitting Criterion.

	*R* ^2^	Adjusted *R* ^2^	Predictive *R* ^2^	Model p-value
(A) Difference in ICII Ratio	0.86	0.83	0.68	< 0.001
(B) Difference in Effectiveness Index	0.36	0.12	-0.35	0.284
(C) Difference in Average CGM - Anticipatory	0.81	0.71	0.06	0.011
(D) Difference in Average CGM - Exercise	0.66	0.47	-0.11	0.075
(E) Difference in Slope of CGM - Anticipatory	0.85	0.77	0.62	0.005
(F) Difference in Slope of CGM - Exercise	0.45	0.24	-1.43	0.170

The ICII was also among the most impactful factors in all models of related to glycemia (**Figures 4C-F**). The variables with the most significant relationship to the difference in effectiveness index include the age, BMI, and duration of diabetes (**Figure 4B**); although, the total percentage of variance in effective index that was explained by this model was relatively low (**Figure 3B**) and the corresponding MLR model performed poorly in all model fitting criteria assessed (**Table 4B**). The calculated effectiveness index was for the anticipatory period prior to the race was large and negative, indicating a higher glucose concentration than was estimated for that time. The difference in effectiveness index indicated a higher difference on race day than on training, which may indicate a trend towards a higher insulin requirement prior to the race (p = 0.005). This elevation in required insulin on the day of the race may be influenced by BMI, relative glycemic control (HbA1c), duration of diabetes, and the observation of the impact may be more notable with an increase in the difference in injected insulin relative to carbohydrates consumed between the race and training sessions.

The variables related to the difference in the average CGM for both the anticipatory period and the exercise period include age, BMI, duration of diabetes, and ICII (**Figures 4C, D**). The MLR models for the difference in average CGM described a high degree of variance in the training data set, but performed poorly in the leave-one-out cross validation of the predictive *R* ^2^ (**Tables 4C, D**).

The difference in the slope of the CGM in the anticipatory period was related to the STAI trait score, the duration of diabetes, HbA1c, and ICII (**Figure 4E**). The MLR model for the difference in the CGM slope in the anticipatory period found the duration of diabetes, the individual personality trait for anxiety-proneness as measured by the STAI-trait survey, and the ICII to be significant variables (**Table 3E**). This model explained a high degree of variance in the data set (*R* ^2^=0.85 and adjusted *R* ^2^=0.77) and would likely describe the variance observed in a broader population of recreational athletes with T1D due to the high predictive *R* ^2^=0.62 (**Table 4E**). In the exercise period, the slope of the CGM had the most significant relationship to the ICII and HbA1c (**Figure 4F**); however, the corresponding MLR model performed poorly in all model fitting criteria assessed (**Table 4F**).

Individuals with longer duration of diabetes were more likely to increase their insulin carbohydrate ratio on the competitive race day than on the non-competitive training day while the reverse was true for those with a shorter duration of diabetes with T1D (r = -0.57, p = 0.05). While there are some significant factors in the difference in the carbohydrate to insulin ratio between the race and training sessions, the absolute amount of carbohydrates consumed prior to the exercise session is not significantly related to the factors under study. In an assessment of carbohydrates consumed during the exercise sessions, it was observed that the athletes competing shorter races (5k or 10k) did not consume any carbohydrates during the exercise sessions. The marathon runners consumed 17-48 g carbohydrates per hour of the race with two participants consuming carbohydrates only when nearing hypoglycemia and the other 4 participants consuming carbohydrates on a pre-determined schedule. In the 6 marathon participants, the additional carbohydrate supplementation began 30 minutes or later into the race.

The Empatica E4 wristband was worn by 4 individuals with successful data collection in both the training and athletic competition sessions. There was no significant difference in estimated energy expenditure (17, 18) between the two exercise sessions (p = 0.97, confidence interval: [-1.82, 1.79]). The difference in galvanic skin response (GSR) between the race and training sessions was highly negatively correlated with the difference in the effectiveness index estimate (r = -0.83, p = 0.10). The larger increase in GSR in the race over training corresponded with a larger negative effectiveness index (higher insulin requirement) on race day though the relation was not statistically significant. The heart rate difference between exercise sessions was correlated with the difference in the average CGM (r = 0.89, p = 0.08), though again not statistically significant.

## 4 Discussion

Elevations in blood glucose concentration and reductions in insulin sensitivity prior to and during an athletic competition relative to exercise in training at the same intensity have been observed in several recreational athletes with T1D. Competition stress may lead to an elevated glucose trend when compared to a training exercise session at the same intensity. This elevation is primarily due to an increasing glucose trend observed in the period of time prior to the competition when the individual is making treatment decisions for the upcoming race and may be experiencing anticipatory stress. This increasing CGM slope prior to the competition may be influenced by the individual’s anxiety-proneness as measured by the STAI-trait survey, duration of diabetes, diabetes management as measured by HbA1c, and the insulin dosing behavior as measured by the ICII (**Table 3E**).

With an increasing duration of diabetes, the individual may be expecting this increase in glucose concentration with the athletic competition and take preventative measures to avoid race-day hyperglycemia such as injecting more insulin with any carbohydrates consumed pre-race. Despite these corrective actions, the average CGM was higher in the first 30 minutes of exercise on the competitive race day compared to the non-competitive session in many of the subjects. The increase in ICII may also relate to a desire for euglycemia with a plan to consume mid-race nutrition to maximize performance during an endurance event. The exercise management in T1D consensus statement and the guidelines for competitive athletes with T1D (1, 2) recommend consumption of 60 to 90g of carbohydrate per hour to maximize performance in events of duration similar to a marathon. Our marathon participants in this study consumed between 17-48 g of carbohydrates per hour of the race. Additional counseling may be required to encourage athletes to consume more carbohydrates to maximize performance while ensuring confidence in the higher insulin administration required to maintain euglycemia. While the time period compared between the competition and non-competition exercise sessions are of the same duration in this analysis, the total duration of exercise in the competition exercise sessions are frequently longer (i.e. the participants completing a marathon distance did not complete this duration of activity in the non-competition session). This may have influenced the individual treatment strategies regarding ICII.

Fear of hypoglycemia may also alter the ICII as participants may have aimed for higher CGM profiles in training or on race day. As many of the more newly-diagnosed individuals utilized a higher ICII, taking less insulin on race day, which led to higher BGC, additional education for these individuals may be recommended to ensure a similar level of confidence in taking the required larger insulin dose prior to exercise on the day of an athletic competition. As practiced by more experienced racers with T1D, increasing or maintaining the insulin dose compared to the dose on the non-competition day was more appropriate. A concern regarding hypoglycemia or the need to cease running while treating a hypoglycemic event is reasonable as it may be detrimental towards meeting the individual goals set for the athletic competition. A deeper assessment between the behaviors of the athletes with T1D and concerns regarding hypoglycemia is of interest.

The carbohydrate-insulin ratio is a static value determined by the person with diabetes and the doctor regarding the amount of insulin to give for an amount of carbohydrates at a particular time of the day. The ingested-carbohydrate-to-injected-insulin (ICII) ratio was calculated as the specific amount of insulin administered, above the basal infusion rate, relative to the reported amount of carbohydrates consumed. This metric aimed to capture the individual treatment strategies for the competition and non-competition exercise sessions. The participants in this study are experienced in managing their diabetes while exercising, and in all cases the ICII was larger than the reported carbohydrate-insulin ratio, a relative reduction in insulin dose for the exercise period, as is recommended (1).

All the participants in this study had experience running races with T1D and had a personal CGM with a prescription from their medical care team. Our study team did not provide any training regarding diabetes management, or the exercise guidelines outlined in the consensus statements. The participants in this study appeared to prioritize avoiding hypoglycemia with the trade-off of spending a greater percentage of time in mild hyperglycemia on both the day of the athletic competition and the non-competition exercise session compared with the recommended guideline of >70% between 70-180mg/dL and <25% for glucose levels greater than 180mg/dL (2). The complete avoidance of hypoglycemia during exercise, 0% of time <70mg/dL for all subjects, indicates skillful use of the CGM and the CGM trends in combination with timely supplementation of carbohydrates when required. Due to the desire to not cease exercise as is recommended during a hypoglycemic event (19), this study participants appeared to be highly proactive in preventing hypoglycemia with additional carbohydrate consumption prior to the start of the exercise session if the CGM trend indicated a hypoglycemia risk in their opinion. This is likely common in this population that would be hesitant to stop exercising when working towards a competition goal.

The degree to which each person cared about their performance in a race and the corresponding degree of athletic competition stress experienced has not been quantified. We aimed to focus solely on competitions with meaningful, personal value, but that interpretation will be dependent on the individual. Despite that limitation and our small sample size (10 participants completing 12 competitions and 12 exercise-intensity matched non-competition exercise sessions), trends in the impact of competition stress have been observed. Due to the small sample size, the data was not separated into a training and testing set. The predictive *R* ^2^ may allow for an assessment of overfitting to the available data. The models for the difference in the ICII ratio and the difference in the slope of the CGM in the anticipatory period both yield high predictive *R* ^2^ values. The trends observed in this study should be viewed as a preliminary observation and further research with a larger sample is necessary to confirm the findings of this study.

Competition stress frequently leads to an elevated glucose trend when compared to a training session at the same intensity. The elevation in glycemia is impacted by the individual behavior related to ingested carbohydrate to injected insulin ratio between the race and training session. With an increasing duration of diabetes, the individual may be expecting this phenomenon and take preventive measures such as administering more insulin with any carbohydrates consumed pre-race. Additional education for the newly-diagnosed individuals may be valuable for reducing hyperglycemia on the day of the athletic competition to help athletes reach their peak potential. The effectiveness index, which accommodates both the behavioral impact of the ingested carbohydrate to injected insulin ratio and the impact on CGM, indicated a general trend towards higher insulin requirement during the athletic competition with the trend related to several demographic traits.

## Data Availability Statement

The raw data supporting the conclusions of this article will be made available by the authors, without undue reservation.

## Ethics Statement

The studies involving human participants were reviewed and approved by Illinois Institute of Technology Institutional Review Board. The patients/participants provided their written informed consent to participate in this study.

## Author Contributions

NH developed the study protocol, conducted the research study, and conducted the primary data analysis. RB assisted in development of the study protocol and conducting the research study. She also provided feedback on the analysis and interpretation of the results. SM contributed to the data analysis. MS assisted in conducting the research study and participated in the data analysis. MR participated in the data analysis and interpretation of the results. LQ assisted in the development of the study protocol and provided data analysis and interpretation of the results. AC assisted in the development of the study protocol and provided data analysis and interpretation of the results. All authors contributed to the article and approved the submitted version.

## Funding

This work is sponsored by NIH NIDDK under grants 1DP3DK101075 and F31DK116524, and JDRF under grant 2-SRA-2017-506-M-B made possible through collaboration between the JDRF and The Leona M. and Harry B. Helmsley Charitable Trust.

## Conflict of Interest

The authors declare that the research was conducted in the absence of any commercial or financial relationships that could be construed as a potential conflict of interest.

## Publisher’s Note

All claims expressed in this article are solely those of the authors and do not necessarily represent those of their affiliated organizations, or those of the publisher, the editors and the reviewers. Any product that may be evaluated in this article, or claim that may be made by its manufacturer, is not guaranteed or endorsed by the publisher.
