# Supplementary material for: Observational Study of Glycemic Impact of Anticipatory and Early-Race Athletic Competition Stress in Type 1 Diabetes
Source: Front Clin Diabetes Healthc. 2022 May 3;3:816316. doi: 10.3389/fcdhc.2022.816316 (PMC10012106; doi:10.3389/fcdhc.2022.816316)
Supplement: Supplementary file 1 [file DataSheet_1.pdf]

Supplementary Material

1 SUPPLEMENTARY TABLES

Survey	Study Participant Scores	Population Average
STAI-T	70 % [29,89]	50 % [25, 75]
SCAT	22 [19,24]	> 17 for low, <24 for high
HFS-B	21 [18, 24]	19.9 ± 9.3
HFS-W	19 [16, 24]	22.3 ± 14.4

Table S1. Survey Response scores for Study Participants. STAI-T: State Trait Anxiety Inventory - Trait Score, SCAT: Sport Competition Anxiety test, HFS-B: Hypoglycemia Fear Survey II - Behavior, HFS-W: Hypoglycemia Fear Survey II - Worry

Metric	Competition Session	Non-Competition Session	p-value
Average Exercise CGM Glucose	178.2 ± 60.3	156.8 ± 68.6	0.28
Slope of CGM in Exercise	1.36 ± 6.07	-2.59 ± 2.68	0.02

Table S2. Difference in CGM metrics between the Competition and Non-Competition Sessions

Response Variable	Percentage of Variance Explained
(A) Difference in ICII Ratio	90.0 %
(B) Difference in Effectiveness Index	38.2 %
(C) Difference in Average CGM - Anticipatory	86.5 %
(D) Difference in Average CGM - Exercise	85.0 %
(E) Difference in Slope of CGM - Anticipatory	84.5 %
(F) Difference in Slope of CGM - Exercise	50.4 %

Table S3. The percentage of response variable variance captured by each PLS model.

2 PROCEDURES OF DATA ANALYSIS

Calculation of the ICII Ratio The ingested carbohydrate to injected insulin ratio describes the insulin dosing behavior in the time period of study. The ingested carbohdrate value is the summation of the total amount of carbohydrates in grams consumed in the time period. The injected insulin value is the total amount of insulin above the standard basal rate in units given as correction doses and as insulin given with carbohydrates in the time period of study. If a basal rate reduction occurred in the time period, the amount of insulin less than the standard basal was subtracted from the injected insulin value. The ratio of the ingested carbohydrate to injected insulin is then compared between the competition and non-competition sessions as a metric describing the diabetes treatment decisions in that time period.

Calculation of the Slope of the CGM For the models of the slope of the CGM for a period of interest, the slope was determined through simple linear regression. All of the CGM samples for the time period of interest were utilized as data points of the independent variable. A single line was fit to the data to determine the slope for the time period.

Calculate of the Rate of Change of the CGM For Figure **??** representing the rate of change of the CGM, each data point for the rate of change was calculated as the difference between the CGM value and the CGM value of the previous time point.

Calculation of the Effectiveness Index Discerning the difference between intrapatient variability in insulin sensitivity and the behavioral differences in lifestyle choices from day to day on the impacted glycemic trend can be a challenge. To analyze the difference between the expected impact of the administered insulin or consumed carbohydrates on the glucose concentration, the concept of the oral carbohydrate net effect has been proposed (Patek et al., 2016). The effectiveness index is the additive inverse of the summation of the net effect estimates over a period of time as a surrogate metric for insulin sensitivity.

Of all factors impacting the glycemic trends, the oral carbohydrate consumption is considered to be the most impactful. As such, the meal arrival process is estimated from retrospective data of the CGM trend and the insulin delivery data. The glycemic response to a meal will be highly impacted by the current insulin sensitivity so the estimated meal effect will be higher in times of relative insulin resistance or lower in times of relative insulin sensitivity. As such, the effectiveness index has been developed to summarize the daily relative insulin sensitivity as determined from the net effect index (Ozaslan et al., 2018).

The net effect core algorithm as described by Patek et al. (Patek et al., 2016) is as follows. The goal of the net effect algorithm is to compute the estimate for a vector of meal inputs such that the error between the filtered CGM and the predicted glucose concentration from the subcutaneous oral glucose minimal model (SOGMM) is minimized. This relies upon the core two-compartment minimal model (Equations S1-S2) (Bergman et al., 1979) to capture the glucose-insulin kinetics

$$\dot{G}(t) = -\left(S_g + X(t)\right) \cdot G_b + \left(\frac{R_a(t)}{V_G}\right) \tag{S1}$$

$$\dot{X}(t) = -p_2 \cdot X(t) + p_2 \cdot S_I \left(I(t) - I_b\right) \tag{S2}$$

In Equation S1, $\dot{G}(t)$ represents the plasma glucose concentration, S_g, the fractional glucose effectiveness, $R_a(t)$, the rate of glucose appearance from oral absorption, V_G, the glucose volume of distribution, and G_b, the steady-state or basal glucose concentration for the model. The value for G_b is a personalized parameter and is estimated from the HbA1c value as shown in Equation S3.

$$G_b = \text{HbA1c} \cdot 28.7 - 46.7 \tag{S3}$$

In Equation S2, $\dot{X}(t)$ represents the insulin in the remote compartment, $I(t)$, represents the plasma insulin concentration, I_b, represents the basal or steady-state value for the plasma insulin for the individual in a unfed state, p_2, represents the rate constant for elimination from the remote compartment, and S_I, the insulin sensitivity parameter represents the ability of insulin to influence glucose production and glucose utilization.

The relationship between plasma glucose concentration, $G(t)$, and the subcutaneous glucose concentration, $G_{\text{CGM}}(t)$, is modeled as a first order delay as shown in Equation S4. The parameter, k_{sc}, represents both the physiological delay associated with diffusion and the CGM sensor delay.

$$\dot{G}_{\text{CGM}}(t) = -k_{sc} \left(G_{\text{CGM}}(t) - G(t)\right) \tag{S4}$$

The absorption of oral carbohydrates is modeled through a two-compartment chain, $\dot{D}_1(t)$ and $\dot{D}_2(t)$, as shown in Equations S5 - S6 with k_{abs} and k_τ both representing the rate of oral absorption, and $\omega(t)$ representing the rate of oral carbohydrates consumed and available for absorption at each time, t. The rate of glucose appearance $R_a(t)$ to the plasma glucose (Equation S1) is scaled by the fraction absorbed in the intestine, f, and the body weight of the subject, BW.

$$\dot{D}_1(t) = -k_\tau \cdot D_1(t) + \omega(t) \tag{S5}$$

$$\dot{D}_2(t) = -k_{abs} \cdot D_2(t) + k_\tau \cdot D_1(t) \tag{S6}$$

$$R_a(t) = \frac{D_2(t) \cdot k_{abs} \cdot f}{\text{BW}} \tag{S7}$$

The transport of the subcutaneously infused insulin to the plasma is a three compartment chain with I_{sc1} and I_{sc2} representing the insulin in the interstitial fluid as shown in Equations S8-S10 with $U(t)$ the insulin infusion rate and k_d and k_{cl} representing the rate constants for elimination from the interstitial space and plasma, respectively.

$$\dot{I}_{sc1}(t) = -k_d \cdot I_{sc1}(t) + U(t) \tag{S8}$$

$$\dot{I}_{sc2}(t) = -k_d \cdot I_{sc2}(t) + k_d I_{sc1}(t) \tag{S9}$$

$$\dot{I}_p(t) = -k_{cl} \cdot I_p(t) + k_d I_{sc2}(t) \tag{S10}$$

The SOGMM is linearized around the basal plasma insulin I_b and basal glucose concentration G_b to obtain the linear, time invariant model (Equations S11-S12)

$$\dot{x}(t) = A_c x_c(t) + B_c u_c(t) + G_c \omega(t) \tag{S11}$$

$$y_c(t) = C_c x_c(t) \tag{S12}$$

with the state vector

$$x(t) = [\partial G(t),\, \partial X(t),\, \partial I_{sc1}(t),\, \partial I_{sc2}(t),\, \partial I_p(t),\, \partial G_{sc}(t),\, \partial D_1(t),\, \partial D_2(t)]' \tag{S13}$$

and the corresponding state matrices:

$$A_c = \begin{bmatrix} -S_g & -G_b & 0 & 0 & 0 & 0 & 0 & \frac{k_{abs} \cdot f}{BW \cdot V_G} \\ 0 & -p_2 & 0 & 0 & \frac{p_2 \cdot S_I}{V_I \cdot BW} & 0 & 0 & 0 \\ 0 & 0 & -k_d & 0 & 0 & 0 & 0 & 0 \\ 0 & 0 & k_d & -k_d & 0 & 0 & 0 & 0 \\ 0 & 0 & 0 & k_d & -k_{cl} & 0 & 0 & 0 \\ k_{sc} & 0 & 0 & 0 & 0 & -k_{sc} & 0 & 0 \\ 0 & 0 & 0 & 0 & 0 & 0 & -k_\tau & 0 \\ 0 & 0 & 0 & 0 & 0 & 0 & k_\tau & -k_{abs} \end{bmatrix} \tag{S14}$$

$$B_c = [0\,0\,1\,0\,0\,0\,0\,0]', \quad G_c = [0\,0\,0\,0\,0\,0\,1\,0]', \quad C_c = [0\,0\,0\,0\,0\,1\,0\,0]'. \tag{S15}$$

The only patient specific parameters are the bodyweight, BW, and the insulin senstivity value, S_I, which is estimated based upon their individual insulin requirements

$$S_I = \exp(-6.4417 - 0.063546 \cdot TDI + 0.057499 \cdot TDI_{\text{basal}}) \tag{S16}$$

The model is then discretized using zero-order hold on the inputs and a sample time of $Ts = 5$. The entire state vector can be described as the relationship between the initial state and the output estimate of glucose, $\tilde{y}$, as follows:

$$\tilde{y} = [y(0),\, y(1),\, \ldots,\, y(T)] = A_l x(0) + B_l \tilde{u} + G_l \tilde{\omega} \tag{S17}$$

where A_l, B_l, G_l are defined as

$$A_l = \begin{bmatrix} C \\ CA \\ CA^2 \\ \vdots \\ CA^T \end{bmatrix} \quad B_l = \begin{bmatrix} 0 & 0 & \ldots & 0 \\ CB & 0 & \ldots & 0 \\ CAB & CB & \ldots & 0 \\ \vdots & \vdots & \ddots & \vdots \\ CA^{T-1}B & CA^{T-2}B & \ldots & CB \end{bmatrix}$$

$$G_l = \begin{bmatrix} 0 & 0 & \ldots & 0 \\ CG & 0 & \ldots & 0 \\ CAG & CG & \ldots & 0 \\ \vdots & \vdots & \ddots & \vdots \\ CA^{T-1}G & CA^{T-2}G & \ldots & CG \end{bmatrix} \tag{S18}$$

The net effect estimate is then composed of a linear combination of basis vectors, $e_j = [11]$, and

$$\hat{\omega} = \sum_{j=1}^{n} \vartheta_j e_j = E\vartheta \tag{S19}$$

where ϑ is the solution of the following optimization problem

$$\min_{x(0),\vartheta} \big[(\xi - A_l x(0) - G_l E\vartheta)' \Lambda_{fit} (\xi - A_l x(0) - G_l E\vartheta) + x(0)' \Lambda_{reg,1} x(0) + \vartheta' E' \Lambda_{reg,2} E\vartheta\big] \tag{S20}$$

with ξ representing the difference between the scaled CGM value and the modeled insulin pharmacodynamics as follows

$$\xi = (CGM - G_b) - B_l \tilde{u} \tag{S21}$$

and as such, the minimization provides a net effect input vector, ϑ, that describes all variability present in the CGM which is not explained by the plasma insulin pharmacodynamics. The net effect value, ω, is positive when the glucose concentration is above the SOGMM glucose with only insulin as an input and is negative when the glucose concentration is less. An extension to this net effect estimate is proposed in this work, such that the known carbohydrate consumption is included. The interpretation of the net effect estimate is then such that a positive value is above the expectation for the meal absorption profile relative

to the insulin effect. This is completed through a redefinition of ξ as follows

$$\xi = (CGM - G_b) - B_l\tilde{u} - G_l\tilde{\omega}_c \tag{S22}$$

where $\tilde{\omega}_c$ is a vector of the the amount of carbohydrates consumed and the with the optimization problem unchanged (Equation S20).

The effectiveness index is the additive inverse of the summation of the net effect estimates over a period of time as shown in Equation S23.

$$E_I = -\hat{\omega} = \sum_{k=1}^{n} \vartheta_k e_k \tag{S23}$$

For the sake of intuition, the E_I is defined as the additive inverse such that a positive value represents and increased insulin sensitivity (lower glucose concentration than estimated) and a negative E_I indicates reduced insulin sensitivity or relative insulin resistance. Ozaslan et al. (Ozaslan et al., 2018) considered the period of interest to be a 24-hour calendar day. The E_I was calculated for the exercise anticipatory period (3 hours) with the net effect vector containing samples for 8 hours prior and 4 hours after the period of study to reduce the influence of edge effects.

REFERENCES

Bergman, R. N., Ider, Y. Z., Bowden, C. R., and Cobelli, C. (1979). Quantitative estimation of insulin sensitivity. *Am J Physiol* 236, E667–77

Ozaslan, B., Patek, S. D., Grabman, J. H., Shepard, J. A., Dassau, E., Breton, M. D., et al. (2018). Body Mass Index Effect on Differing Responses to Psychological Stress in Blood Glucose Dynamics in Patients With Type 1 Diabetes. *J Diabetes Sci Technol* 12, 657–664. doi:10.1177/1932296818758103

Patek, S. D., Lv, D., Ortiz, E. A., Hughes-Karvetski, C., Kulkarni, S., Zhang, Q., et al. (2016). Empirical Representation of Blood Glucose Variability in a Compartmental Model. In *Predict Methods Blood Glucose Conc* (Springer, Cham). 133–157. doi:10.1007/978-3-319-25913-0_8
